# Thermal diffusivity measurement for urchin-like gold nanofluids with different solvents, sizes and concentrations/shapes

**DOI:** 10.1186/1556-276X-7-667

**Published:** 2012-12-06

**Authors:** Gerardo A López-Muñoz, José Abraham Balderas-López, Jaime Ortega-Lopez, José A Pescador-Rojas, Jaime Santoyo Salazar

**Affiliations:** 1 Química Aromática SA, Río Grande S/N, Col. Santa Catarina, Acolman, Mexico State, CP 55875, Mexico; 2 UPIBI-IPN, Av. Acueducto S/N, Col. Barrio la Laguna Ticomán, Mexico City, CP, 07340, Mexico; 3 Biotechnology and Bioengineering Department, CINVESTAV, Av. IPN 2508, Col. San Pedro Zacatenco, Mexico City, CP, 07360, Mexico; 4 ICGDE-BUAP, 4 Sur 104 Edificio Carolino, Col. Centro Histórico, Puebla, CP, 72000, Mexico; 5 Physics Department, CINVESTAV, Av. IPN 2508, Col. San Pedro Zacatenco, Mexico City, CP, 07360, Mexico

**Keywords:** Urchin-like gold nanoparticles, Nanofluids, Photopyroelectric, Thermal diffusivity

## Abstract

The thermal properties of nanofluids are an especially interesting research topic because of the variety of potential applications, which range from bio-utilities to next-generation heat-transfer fluids. In this study, photopyroelectric calorimetry for measuring the thermal diffusivity of urchin-like colloidal gold nanofluids as a function of particle size, concentration and shape in water, ethanol and ethylene glycol is reported. Urchin-like gold nanoparticles were synthesised in the presence of hydroquinone through seed-mediated growth with homogeneous shape and size ranging from 55 to 115 nm. The optical response, size and morphology of these nanoparticles were characterised using UV-visible spectroscopy and transmission electron microscopy. The thermal diffusivity of these nanofluids decreased as the size of the nanoparticles increased, and the enhancement depended on the thermal diffusivity of the solvent. The opposite effect (increase in thermal diffusivity) was observed when the nanoparticle concentration was increased. These effects were more evident for urchin-like gold nanofluids than for the corresponding spherical gold nanofluids.

## Background

Currently, thermal properties of nanofluids, i.e. mixtures of nanomaterials suspended in an organic or inorganic base fluid, are intriguing because of their various uses, which range from bio-applications to the next generation of heat-transfer fluids. For example, gold nanofluids are a promising material for bio-applications because of their biocompatibility and thermo-optical properties. In particular, urchin-like gold fluids have a higher surface area that endows them with specific catalytic and thermo-optical qualities [1]. The surface plasmon resonance of urchin-like gold nanofluids can be tuned to the near-infrared region by increasing the particle size, which makes the particles potentially effective in photothermal therapy and as contrast agents for diagnosis [2]. In addition, local electromagnetic field enhancement at the tips of branched particles and thermal conductivity enhancement make these materials a candidate for application in electromagnetic hyperthermia therapy [3,4]. Thermal studies of nanofluids have mainly focused on thermal conductivity measurements, but recently, other techniques have been developed for thermal diffusivity measurements in nanofluids, such as the hot-wire technique and thermal lens spectrometry [5-7].

In the present study, the aqueous synthesis of urchin-like gold nanoparticles in the presence of hydroquinone through a seed-mediated growth process is presented. The thermal diffusivity in urchin-like gold nanofluids as a function of particle size (55 to 115 nm) and concentration in different solvents was investigated by photopyroelectric calorimetry. A comparative study of thermal diffusivity in urchin-like and spherical gold nanofluids with approximately equal particle sizes as a function of concentration is also reported. These results will open new horizons for thermal studies of nanofluids for bio-applications and heat-transfer applications.

## Methods

### Hydroquinone synthesis of urchin-like gold nanoparticles

Urchin-like gold nanoparticles are commonly synthesised through a self-seeding growth process, where larger particles grow through the deposition of smaller seeds that form from the epitaxial deposition of atoms. The reduction of gold chloride on the nanoparticle seeds (gold nanoparticles, <20 nm) paired with hydroquinone as a selective reducing agent improves the homogeneous size and shape distribution of the urchin-like particles through physicochemical effects [8,9].

Gold nanoparticle seeds were synthesised by sodium citrate reduction. For this process, a solution of gold chloride is brought to boil, whereupon a solution of sodium citrate is immediately added. The solution is then removed from the heat source once nanoparticle maturation is completed, as indicated by the colour transition.

Urchin-like gold nanoparticles were synthesised by hydroquinone method using consistent concentrations of gold chloride, sodium citrate and hydroquinone; however, the number of seeds gradually decreased, which resulted in the formation of larger urchin-like gold nanoparticles. Nanofluids with varying particle size were centrifuged at 6,000 rpm for 30 min and re-dispersed in high-performance liquid chromatography (HPLC) water, ethanol and ethylene glycol (EG) at a final concentration of 0.1 mg/ml.

To provide nanofluids with different shapes and concentrations, stock solutions of urchin-like gold nanoparticles and spherical gold nanofluids synthesised by hydroquinone method [9,10] were centrifuged at 6,000 rpm for 30 min. The concentrated solutions were re-dispersed in HPLC water, ethanol and ethylene glycol to obtain different nanoparticle concentrations.

All chemicals used were of analytical grade as obtained from Sigma-Aldrich Corporation (St. Louis, MO, USA) and were used as received; HPLC water was used for the synthesis of all the urchin-like and spherical gold nanoparticles.

### Basic photopyroelectric theoretical scheme

As shown in the literature [11], the photopyroelectric signal in the transmission configuration, assuming that the sample thickness *L* is the only variable (with fixed modulation frequency *f*) in the thermally thick regime of the liquid sample, can be expressed as follows:

(1) $$\mathrm{\delta P}=E\exp\left(-\sigma_{s}L\right),$$

where *E* is a complex constant and *σ*_s_ = (1 + *i*)(*πf*/*α*_s_)^1/2^, with *α*_s_ is the thermal diffusivity of the sample. The amplitude │δΡ│ and phase Ф of this equation can be calculated as follows:

(2) $$\left|\mathrm{\delta P}\right|=\left|E\right|\exp\left(-\sqrt{\frac{\pi f}{\alpha_{s}}}L\right),$$

(3) $$\Phi =\Phi_{0}-\sqrt{\frac{\mathrm{\pi f}}{\alpha_{s}}}L.$$

The signal phase Ф is a linear function of the sample thickness, and slope *B* is given by the following:

(4) $$B={\left(\frac{\mathrm{\pi f}}{a_{s}}\right)}^{\frac{1}{2}},$$

from which the thermal diffusivity of the sample can be determined.

### Experimental setup

**Figure 1:**
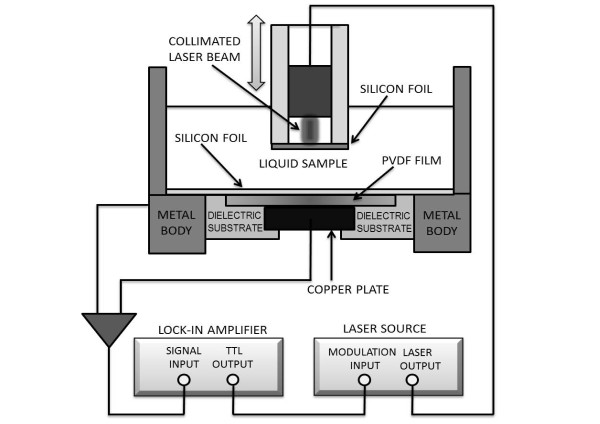
Cross-sectional view of the photopyroelectric experimental system.

A transversal section of the photopyroelectric experimental setup for thermal diffusivity measurements is shown in Figure 1. The photopyroelectric sensor consisted of a PVDF film (25-μm thick) with metal electrodes (Ni-Al) on both sides of a stainless steel body. A silicon foil was attached to the top surface of the photopyroelectric sensor to prevent any possible damage that could result from exposure to the liquid environment. The resultant pyroelectric signals were processed by a lock-in amplifier (model SR830; Stanford Research, Menlo Park, CA, USA) for amplification and de-modulation; the transistor-transistor logic output of the lock-in was used for the modulation control of a 660-nm model laser diode system (IFLEX-2000; Qioptiq Photonics Ltd., Hamble, Hampshire, UK) at a fixed modulation frequency of 1 Hz.

The pyroelectric signal was recorded as a function of the sample thickness by measuring 20 experimental points from a relative sample thickness *l*_*0*_, at 10-μm intervals using a micro-linear stage (model T-LSM025A; Zaber Technologies, Inc., Vancouver, Canada). Linear fits were performed for the pyroelectric phase to obtain the *B* parameter, as defined in the ‘Basic photopyroelectric theoretical scheme’ section, from which the thermal diffusivity of the sample was obtained by means of the relationship *α*_s_ = *π*/*B*^2^. Measurements were performed at room temperature, or 22 ± 2°C.

## Results and discussion

### Nanoparticle characterization

**Figure 2:**
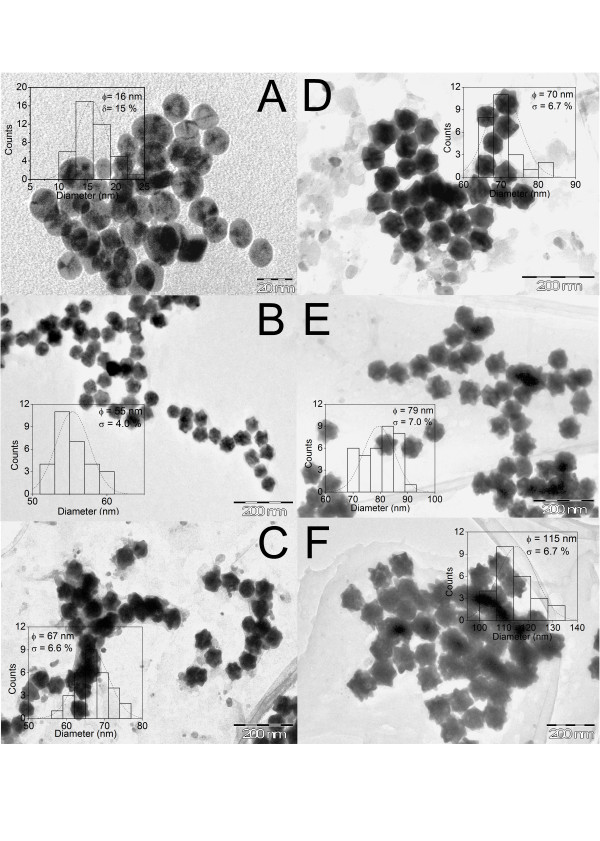
**Representative TEM images and size distributions of the nanoparticle seeds and the urchin-like nanoparticles obtained.** (**A**) 16 nm (seeds), (**B**) 55 nm, (**C**) 67 nm, (**D**) 70 nm, (**E**) 79 nm, (**F**) 115 nm.

The particle size, shape/morphology and nanoparticle distribution in the base medium were determined by transmission electron microscopy (TEM) using a JEOL JEM2010 (JEOL Ltd., Akishima, Tokyo, Japan) operating at 200 kV with a beam current of 105 μA. The TEM specimens were prepared by adding 50 μl of the stock nanoparticle solution onto 200-mesh carbon-coated copper grids. In Figure 2, the TEM images show the larger urchin-like gold nanoparticles that were synthesised by decreasing the number of nanoparticle seeds, as was expected. The dispersion of the urchin-like gold nanoparticles had a median size distribution of nearly 6% for all of the samples, including the large-diameter nanoparticles. This technique is advantageous over single-step methods that use silver ions to increase the facet selectivity during synthesis of the urchin-like nanoparticles; these methods are also less capable of continuously controlling the diameter of branched particles and spherical particles, which is another important method used to tailor the plasmon resonance absorption [8].

**Figure 3:**
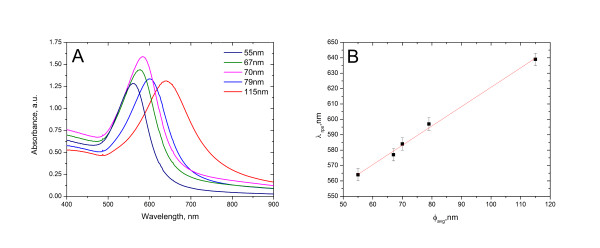
**UV-vis absorption spectrum and shift in the absorption of urchin-like gold nanoparticles.** (**A**) UV-vis absorption spectrum of the synthesised urchin-like gold nanoparticles. (**B**) Shift in the absorption (*λ*_rps_) of urchin-like gold nanoparticles with particle size.

The absorption spectra of the synthesised nanoparticles were measured after the HPLC water background was established using a UV-visible (vis) spectrophotometer (8453 Agilent Technologies, Inc., Santa Clara, CA, USA). Figure 3A shows the UV-vis absorption spectra of the different-sized urchin-like gold nanofluids. As expected, increases in the diameter of the gold nanoparticles resulted in larger absorption (*λ*_max_) values (Figure 3B) according to Mie theory [12,13]; these results provide further evidence that the hydroquinone growth method allows superior tuning of surface plasmon resonance for urchin-like gold nanoparticles.

### Thermal diffusivity measurement

**Figure 4:**
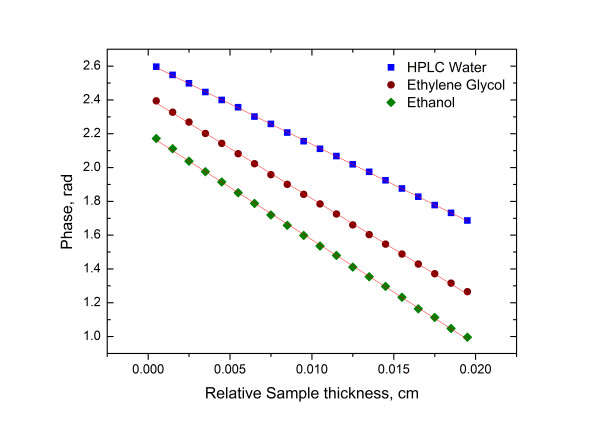
**Typical photopyroelectric curves as a function of sample thickness for HPLC water, ethylene glycol and ethanol.** The continuous lines represent the best linear fits.

The plots in Figure 4 show the signal phase as a function of sample thickness for the solvents used. The recorded thermal diffusivity values were 14.29 × 10^−4^ ± 0.03 × 10^−4^ cm^2^·s^−1^, 9.25 × 10^−4^ ± 0.03 × 10^−4^ cm^2^·s^−1^ and 8.32 × 10^−4^ ± 0.03 × 10^−4^ cm^2^·s^−1^ for HPLC water (Sigma-Aldrich Corporation), EG and ethanol (Sigma-Aldrich Corporation), respectively. The measurements were obtained by utilising the photopyroelectric system as a reference sample for system characterisation with a coefficient of variation within 1% of similar values reported in the literature. The resulting thermal diffusivity values for colloidal urchin-like gold nanoparticles with different sizes in water, ethanol and ethylene glycol are summarised in Table 1. The thermal diffusivity values of colloidal urchin-like and spherical gold nanofluids of two different particle sizes at different concentrations in water, ethanol and ethylene glycol are summarised in Tables 2 and 3. All of the values reported are averaged from ten measurements for each sample, and the standard deviation was calculated as an estimation of uncertainty.

**Table 1:** Thermal diffusivity of urchin-like gold nanofluids for different nanoparticle diameters and solvents

**Particle size (nm)**	**Water**^**a**^	**EG**^**a**^	**Ethanol**^**a**^
55	14.76	9.83	9.05
67	14.71	9.78	8.93
70	14.68	9.73	8.84
79	14.48	9.49	8.61
115	14.34	9.33	8.44

At a constant nanoparticle concentration of 0.1 mg/ml. ^a^Values are presented as (*α* (10^−4^ cm^2^ s^−1^ ) ± 0.03).

**Table 2:** Thermal diffusivity of urchin-like gold nanofluids at different nanoparticle concentrations and in different solvents

**Concentration (mg/ml)**	**Water**^**a**^	**EG**^**a**^	**Ethanol**^**a**^	**Water**^**b**^	**EG**^**b**^	**Ethanol**^**b**^
0.2	14.79	9.87	9.09	14.36	9.36	8.47
0.4	14.83	9.92	9.15	14.37	9.39	8.51
0.6	14.91	10.01	9.28	14.42	9.45	8.56
0.8	14.98	10.13	9.39	14.46	9.49	8.62
1.0	15.11	10.25	9.56	14.53	9.56	8.71

^a^Values are presented as (*α*_55nm_ (10^−4^ cm^2^ s^−1^) ± 0.03). ^b^Values are presented as (*α*_115nm_ (10^−4^ cm^2^ s^−1^) ± 0.03).

**Table 3:** Thermal diffusivity of spherical gold nanofluids at different nanoparticle concentrations and in different solvents

**Concentration (mg/ml)**	**Water**^**a**^	**EG**^**a**^	**Ethanol**^**a**^	**Water**^**b**^	**EG**^**b**^	**Ethanol**^**b**^
0.2	14.74	9.83	9.05	14.33	9.32	8.44
0.4	14.79	9.87	9.10	14.34	9.34	8.47
0.6	14.84	9.91	9.22	14.37	9.39	8.51
0.8	14.91	10.02	9.32	14.41	9.44	8.57
1.0	14.99	10.13	9.43	14.46	9.50	8.64

^a^Values are presented as (α_55nm_ (10^−4^ cm^2^ s^−1^) ± 0.03). ^b^Values are presented as (α_120nm_ (10^−4^ cm^2^ s^−1^ ) ± 0.03).

#### Nanoparticle size

**Figure 5:**
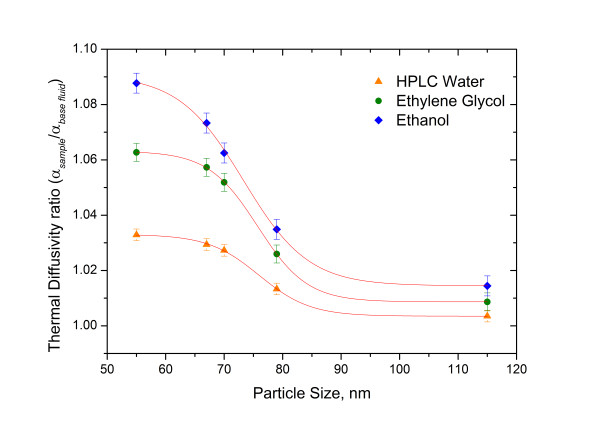
**Increased thermal diffusivity ratio with gold nanoparticle size and different solvents.** The nanoparticle concentration was 0.1 mg/ml.

Figure 5 shows the thermal diffusivity values of urchin-like gold colloid nanofluids as a function of nanoparticle size and in different solvents; the plot also reveals that a constant nanoparticle concentration (0.1 mg/ml) effectively increased the thermal diffusivity ratio (*α*_sample_/*α*_base fluid_) of the nanofluids as the nanoparticle diameter and as the thermal diffusivity of the solvent were reduced. This behaviour can be explained as follows: as the particle size decreases, the surface area-to-volume effective ratio of the particle increases; therefore, the thermal diffusivity and thermal conductivity of the nanofluids are increased. However, for the large-diameter nanoparticles, the surface area-to-volume ratio decreases, and there is consequently no enhancement of thermal diffusivity and thermal conductivity [10,14]. Moreover, ethanol-based nanofluids were observed to have a higher thermal diffusivity ratio than EG- and water-based nanofluids because of their lower thermal diffusivity.

#### Concentration/shape

**Figure 6:**
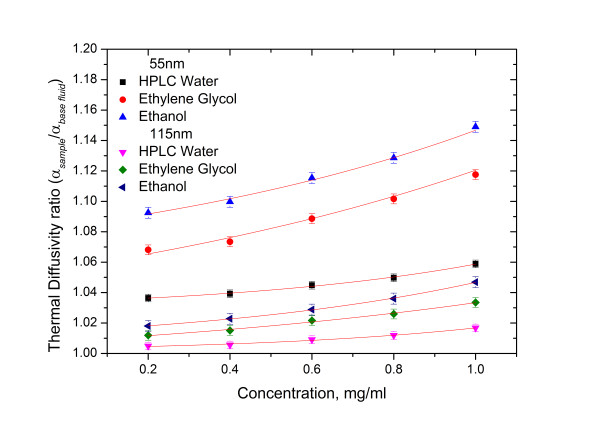
**Thermal diffusivity enhancement ratio of urchin-like gold nanofluids with varying concentrations and solvents.** The average diameters of the nanoparticles were 55 and 115 nm.

Figure 6 shows the dependency of the nanofluid thermal diffusivity on nanoparticle concentration for 55- and 115-nm urchin-like gold nanoparticles; as the nanoparticle concentration increases, the thermal diffusivity ratio (*α*_sample_/*α*_base fluid_) of the nanofluids also increases. Similar results have been reported in the literature for metal nanofluids measured by hot wire method [14,15]. This dependency can be attributed to the volumetric increase of the nanoparticles that effectively decreases the specific heat of the nanofluids; consequently, the thermal diffusivity of the colloidal suspension increases. The enhancement of the surface area-to-volume ratio paired with the decrease in specific heat resulted in an increase in the thermal diffusivity ratio of 55-nm concentrated nanofluids when compared with 115-nm concentrated nanofluids.

**Figure 7:**
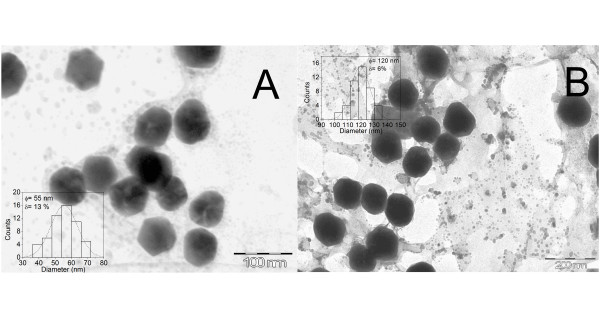
**Representative TEM images and size distributions of the spherical gold nanoparticles obtained.** (**A**) 55 nm and (**B**) 120 nm.

**Figure 8:**
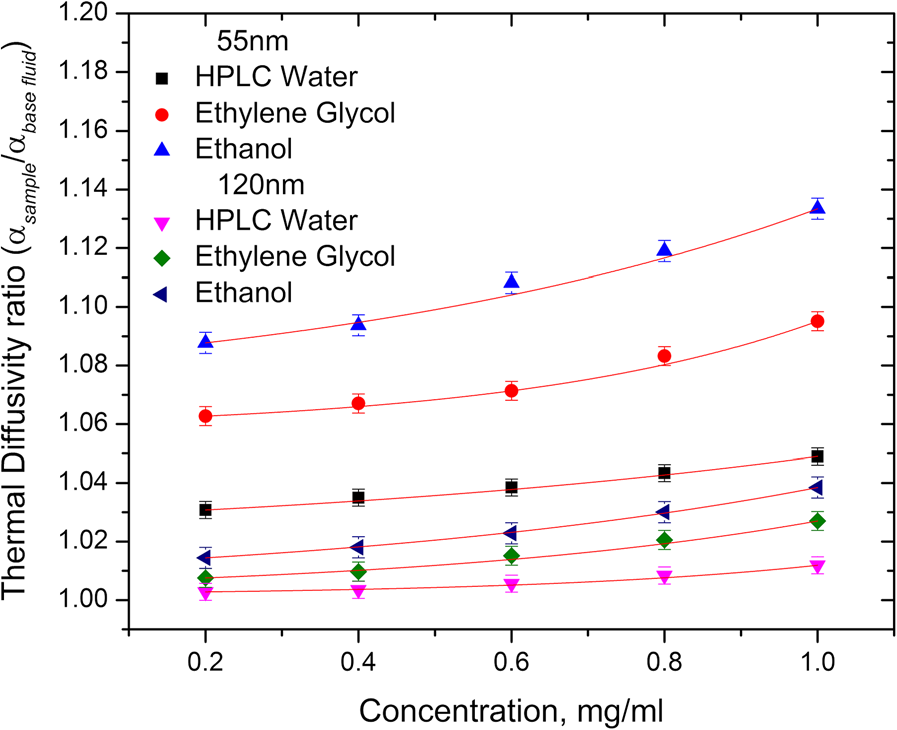
**Thermal diffusivity enhancement ratio of spherical gold nanofluids with varying concentrations and solvents.** The average diameters of the nanoparticles were 55 and 120 nm.

TEM images of spherical gold nanoparticles with sizes similar to those of urchin-like gold nanoparticles are shown in Figure 7. The thermal diffusivity ratio (*α*_sample_/*α*_base fluid_) for 55- and 120-nm spherical gold nanofluids increased as a function of concentration, as shown in Figure 8. However, the thermal diffusivity ratio enhancement was smaller when compared with that of urchin-like gold nanofluids. This dependency can be ascribed to the high surface area of the urchin-like gold nanoparticles when compared with the spherical gold nanoparticles, which exhibit an increase in the surface area-to-volume ratio and decrease in the specific heat. These parameters are more strongly affected by the thermal diffusivity ratio of nanofluids containing urchin-like nanoparticles than by the thermal diffusivity ratio of nanofluids containing spherical nanoparticles.

## Conclusions

The present study investigated the effects of concentration, size and solvent on the thermal diffusivity of urchin-like gold nanofluids prepared by hydroquinone method through seed-mediated growth. The low size dispersion of urchin-like gold nanoparticles synthesised by hydroquinone method is advantageous when compared with single-step methods that are less capable of continuously controlling the diameters of branched particles; such control is critical as it is directly related to the tunable surface plasmon resonance of the urchin-like nanoparticles. The thermal diffusivity ratio changed inversely with nanoparticle size, which varied from 55 to 115 nm for urchin-like gold nanofluids. The thermal diffusivity ratio has been found to increase with nanoparticle concentration and was thus investigated within the range of 1 to 0.2 mg/ml for the gold nanofluids. The thermal diffusivity ratio increased inversely with the thermal diffusivity of the nanofluid solvent. Moreover, the particle shape was found to have an effect on the thermal diffusivity of nanofluids. Experimental data for the thermal diffusivity ratio as a function of concentration and size for gold nanofluids depict similar behaviours in the enhancement ratio when compared with the thermal diffusivity using other techniques reported in the literature with high accuracy. Because of the small amount of sample required (600 μl) and the possibility to provide additional information such as the thermal and optical parameters of the sample with the developed sensor, photopyroelectric calorimetry is a promising alternative to classical techniques for measuring the thermal diffusivity of nanofluids.

## Abbreviations

EG: Ethylene glycol; HPLC: High-performance liquid chromatography; TEM: Transmission electron microscopy; Vis: Visible.

## Competing interests

The authors declare that they have no competing interests.

## Authors’ contributions

GALM prepared all the samples, set up the photopyroelectric method and measured and analysed the photopyroelectric data. JAPR and JSS measured and analysed the UV-vis and TEM data. JOL and JABL designed the experiments and wrote the manuscript. All authors read and approved the final manuscript.
